# Supplementary material for: The Significance of Xylem Structure and Its Chemical Components in Certain Olive Tree Genotypes with Tolerance to Xylella fastidiosa Infection
Source: Plants (Basel). 2024 Mar 23;13(7):930. doi: 10.3390/plants13070930 (PMC11013585; doi:10.3390/plants13070930)
Supplement: Supplementary file 1 [file plants-13-00930-s001.zip › plants-2912039-supplementary.pdf]

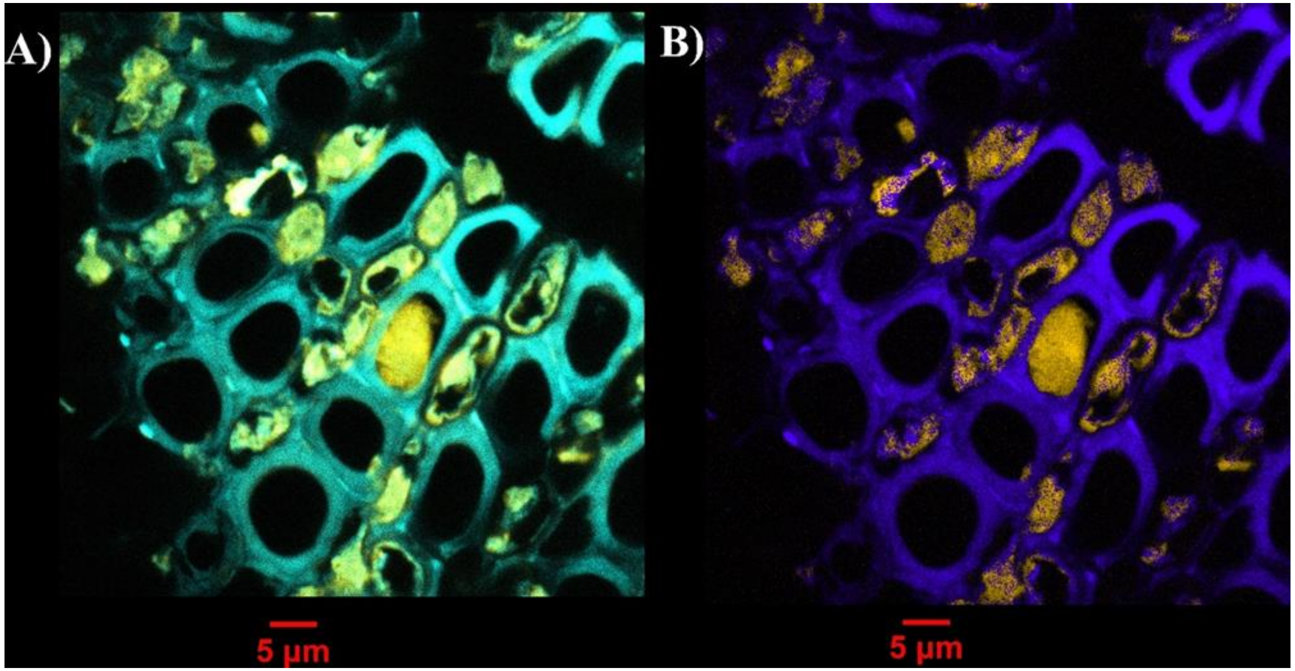

Supplementary Figure S1. A) Representative image of gels and phenolic compounds (yellow signal) associated with the xylem tissue (cyan signal); gels and phenolic compounds are everted in the lumen of the vessel (mechanism that leads to the tylosis formation. **B)** Image of the panel A after Spectral imaging processing coupled with linear unmixing image analysis that allows a more defined fluorescence signals separation (emission fingerprinting).

Supplementary Table S1. Features of the putatively resistant (PRPs) and symptomatic control (CTRLs) plants. UPGMA clustering based on short sequence repeat (SSR) DNA fingerprint is reported (Pavan et al., 2021).

PRP code	Locality	Clustering	***Xf* concentration in the PRPs (cfu/ml)**	***Xf* concentration in the CTRLs (cfu/ml)**	**Syndrome severity in the PRPs**	**Syndrome severity in the CTRLs**
SX32	Muro Leccese	K2**	7.91E + 05	8.63E + 05	0.00	3.00
SX67	Bagnolo del Salento	K1/L*	1.00E + 02	7.30E + 05	0.00	1.50
SX31	Minervino	K1/L*	3.13E + 05	1.00E + 07	0.75	2.25
SX2	Lequile	K1/L*	4.19E + 05	1.00E + 07	0.00	2.00
SX25	Presicce	K1/C*	1.00E + 02	2.37E + 04	0.75	3.00
SX27	Sannicola	K1/C*	1.00E + 02	8.75E + 05	1.00	3.00
SX26	Gallipoli	K1/C*	3.51E + 03	1.09E + 05	0.13	2.75
SX29	Sannicola	K1/C*	4.41E + 03	8.55E + 04	1.25	2.75
SX5	Lequile	K1*	5.15E + 03	5.10E + 04	0.00	2.50
SX33	Cutrofiano	K1*	1.04E + 03	6.43E + 05	0.00	1.50

* **Cluster K1**: genetic cluster grouping most PRPs with 22 Italian cultivars, including the resistant cultivars 'Leccino' and 'FS17'. The cluster K1 includes also the two sub-clusters **K1/L** (genotypes closely related with the cultivar Leccino) and **K1/C** (genotypes closely related with the cultivar Ciciulara).

****Cluster K2**: cluster with several Tunisian cultivars ('Regueb', 'Chemlali Sfax', 'Sayali', and 'Tamri Douiret').
